# The Performance of HepG2 and HepaRG Systems through the Glass of Acetaminophen-Induced Toxicity

**DOI:** 10.3390/life11080856

**Published:** 2021-08-21

**Authors:** Tamás Lőrincz, Veronika Deák, Kinga Makk-Merczel, Dóra Varga, Péter Hajdinák, András Szarka

**Affiliations:** 1 Laboratory of Biochemistry and Molecular Biology, Department of Applied Biotechnology and Food Science, Budapest University of Technology and Economics, Szent Gellért tér 4, H-1111 Budapest, Hungary; lorincz.tamas@vbk.bme.hu (T.L.); deak.veronika@vbk.bme.hu (V.D.); makk-merczelk@edu.bme.hu (K.M.-M.); dora.varga@edu.bme.hu (D.V.); hajdinak.peter@vbk.bme.hu (P.H.); 2 Biotechnology Model Laboratory, Faculty of Chemical Technology and Biotechnology, Budapest University of Technology and Economics, Szent Gellért tér 4, H-1111 Budapest, Hungary; 3 Department of Molecular Biology, Institute of Biochemistry and Molecular Biology, Semmelweis University, H-1428 Budapest, Hungary

**Keywords:** HepG2, HepaRG, toxicology, in vitro model, cell death, hepatocyte

## Abstract

Investigation of drug-induced liver injuries requires appropriate in vivo and in vitro toxicological model systems. In our study, an attempt was made to compare the hepatocarcinoma HepG2 and the stem cell-derived HepaRG cell lines both in two- and three-dimensional culture conditions to find the most suitable model. Comparison of the liver-specific characteristics of these models was performed via the extent and mechanism of acetaminophen (APAP)-induced hepatotoxicity. Investigating the detailed mechanism of APAP-induced hepatotoxicity, different specific cell death inhibitors were used: the pan-caspase inhibitor zVAD-fmk and dabrafenib significantly protected both cell lines from APAP-induced cell death. However, the known specific inhibitors of necroptosis (necrostatin-1 and MDIVI) were only effective in differentiated HepaRG, which suggest a differential execution of activated pathways in the two models. By applying 3D culture methods, CYP2E1 mRNA levels could be elevated, but we failed to achieve a significant increase in hepatocyte function; hence, the 3D cultivation especially in APAP toxicity studies is not necessarily worth the complicated maintenance. Based on our findings, the hepatocyte functions of HepaRG may stand between the properties of HepG2 cells and primary hepatocytes (PHHs). However, it should be noted that in contrast to PHHs having many limitations, HepaRG cells are relatively immortal, having a stable phenotype and CYP450 expression.

## 1. Introduction

The investigation of drug-induced hepatotoxicity at in the center of toxicological studies since drug-induced liver injury is a major cause of late-stage clinical drug attrition, market withdrawal, and acute liver failure [1]. The prediction of clinical drug-induced liver damage is of paramount importance at the earliest possible stage of development.

The most widely used experimental model related to human acute liver failure is the acetaminophen (APAP)-based model [2]. APAP is a commonly used antipyretic and analgesic drug with a large therapeutic window, but at a high dose or in combination with alcohol or other xenobiotics, it causes centrilobular hepatic necrosis, resulting in acute liver failure [3]. APAP overdose is one of the most frequent reasons for acute liver failure in humans, accounting for nearly 50% of all cases [2,4]. Approximately 5–10% of APAP is oxidized by CYP450s (CYP1A2, CYP2E1, and CYP3A4) into the highly reactive metabolite, N-acetyl-p-benzoquinone imine (NAPQI) [5], which is detoxified upon conjugation with glutathione (GSH) [6,7]. It is now believed that the binding of NAPQI to mitochondrial proteins is central in the toxicity of APAP.

Different forms of cell death such as apoptosis, necroptosis, and pyroptosis can play a role in APAP-induced cell death [8]. Our and other research groups recently found that ferroptosis can also be involved in APAP-induced toxicity in primary mouse hepatocytes [9] and in a murine model [10,11]. Hepatoma cell lines such as HepG2, HuH7, and SK-Hep1 are commonly used in vitro toxicological models. They can be characterized by low CYP450 activity, and they typically respond by apoptosis to high doses of APAP treatment [12,13,14]. It was recently described that the activation of autophagy may be beneficial against APAP-induced hepatotoxicity by removing APAP adducts and damaged mitochondria in mouse livers [15]. Finally, some studies also suggest a role for pyroptosis (an inflammatory form of programmed necrosis) in APAP toxicity [16,17].

Although the most valuable data come from studies on primary human hepatocytes (PHHs) [18], they have several limitations. First, it is difficult to obtain human liver tissue in sufficient quantities. Furthermore, the health status, the age of donors, and overall interindividual differences can all influence the experimental results.

As a result, there is a high pressure to replace PHHs in liver-related studies. Strategies to achieve better hepatocyte functions include genetic modification of long-established hepatoma cell lines or improvement of culture conditions, such as the development of cocultures and/or three-dimensional (3D) cultures [19,20,21,22,23,24,25,26,27].

HepG2 is a well-known hepatocarcinoma cell line. On one hand, it can be characterized by low drug-metabolizing capacity and poor hepatic functions due to dedifferentiation. On the other hand, its maintenance is quite easy and cheap. Thus, attempts have been made to preserve its hepatic features by the overexpression of CYP2E1 [28].

Recently, HepaRG cell line has been proposed as a better in vitro model for the investigation of APAP toxicity. This cell line has been established from a liver tumor associated with chronic hepatitis C [29]. HepaRG cells are capable of differentiating into two subpopulations: one with hepatocyte-like morphology and function and another with the appearance of biliary epithelial-like cells [29,30,31]. Hepatocyte-like cells have a characteristic granular appearance and grow in clusters or “hepatocyte islands”. These islands are surrounded by the flatter, clearer biliary epithelial-like cells [32]. Their close resemblance to normal human hepatocytes makes them suitable for many applications, including drug metabolism studies [33,34,35]. Exposure of HepaRG cells to APAP showed liver cell-like features, such as GSH depletion, APAP protein adduct formation, mitochondrial dysfunction, and lactate dehydrogenase release [32].

The main goal of our study was to investigate and compare the toxicological applicability of HepG2 and differentiated HepaRG cell lines maintained in different 2D and 3D cell culture systems. The degree of liver-specific characteristics of these in vitro models were tracked via the extent of APAP-induced hepatotoxicity. We intended to determine the dominance and relationships of various cell death pathways, which, in comparison with those described in human liver tissue, help to establish the most suitable cell line and tissue culture technique for in vitro toxicological studies.

## 2. Materials and Methods

### 2.1. Cell Culture

Cells were grown in a cell culture incubator (Thermo Fisher Scientific, Waltham, MA USA, Thermo Scientific™ Forma™ Series II 3111) at 37 °C, 5% CO_2_, 100% relative humidity.

HepG2 cells were cultured based on ATCC guidelines. Briefly, the cells were maintained in DMEM (Thermo Fisher Scientific, Gibco™) supplemented with 10% FBS (Sigma-Aldrich^®^, St. Louis, MO, USA) and 1% antibiotic/antimycotic (Sigma-Aldrich^®^) (complete growth medium), and they were subcultured before reaching 100% confluence, usually in a 1:4 ratio.

Undifferentiated HepaRG cells were obtained from Biopredic International (Saint-Grégoire, France). The cells were maintained according to the distributor’s guidelines. Briefly, for the differentiation process, cells were seeded homogenously in 96, 24, or 6-well plates (at a seeding density of 9 × 10^3^, 5.5 × 10^4^, or 2 × 10^5^ cells/well, respectively). For the first 14 days, the cells were maintained in William’s E medium (Sigma-Aldrich^®^) containing ADD710C-HepaRG^®^ Growth Medium Supplement with antibiotics (Biopredic) and Glutamax (Gibco™), which was followed by an additional 14-day differentiation phase. The differentiation process was performed in William’s E medium (Sigma-Aldrich^®^) containing ADD720C HepaRG^®^ Differentiation Medium Supplement with antibiotics (Biopredic) and Glutamax (Gibco™). A batch of HepaRG cells used in the experiments thath underwent 14 days of growth and 14 days of differentiation was termed “differentiated HepaRG” throughout this paper.

### 2.2. Three-Dimensional (3D) Cell Cultures

For spheroid cell culture, cells were maintained in Thermo Scientific™ Nunclon™ Sphera™ flasks. For the nanofiber scaffold, 3D cell culture Nanofiber multiwell plates with random oriented nanofibers (Merck, Darmstadt, Germany) were used. APAP treatments were performed on 14-day 3D HepG2 cultures. For HepaRG 3D cultures, the standard 14 + 14-day differentiation process was performed before the experiments.

### 2.3. APAP Treatment of HepG2 and HepaRG Cells

HepG2 cells were seeded homogenously in either 96, 24, or 6-well plates (at a seeding density of 1.5 × 10^4^, 1.5 × 10^5^, 8 × 10^5^ cells/well, respectively). The cells were seeded in complete growth medium and were incubated for 24 h; then, they were replaced with the APAP supplemented complete growth medium for the treatment. The treatment of HepG2 cells with APAP was performed for 24 h.

Twenty-four h before treatment of HepaRG cells, the differentiation medium was replaced by induction medium (William’s E (Sigma-Aldrich^®^) supplemented with ADD650C-HepaRG^®^ Serum-free Induction Medium Supplement with antibiotics (Biopredic) and Glutamax (Gibco™)). Then, treatment of HepaRG cells with APAP was performed for 24 h in induction medium.

For inhibitor profile studies, the APAP supplemented complete growth medium (HepG2) or induction medium (HepaRG) was further supplemented by one of the following agents: zVAD-fmk, Dabrafenib-mesylate, Necrostatin-1, Necrostatin-2, MDIVI-1, α-Tocopherol-acetate, Liproxstatin-1, Ferrostatin-1 (for more details, see Appendix A). Solvent controls were used in all cases for inhibitor profile studies (max. DMSO content, 0.25 *v*/*v*% was applied).

### 2.4. Determination of LC_50_ Values via MTT Assay

Cell viability for LC_50_ curves determination was measured in 96-well plates. Cells were treated as described above. The treatment medium from the plate was discarded and replaced with DMEM (HepG2) or William’s E medium (HepaRG) supplemented with 1/10 volume 5 mg/mL MTT dissolved in PBS. The plate was incubated with the medium supplemented with MTT for 40 min in cell culture incubator; then, it was replaced with dimethyl sulfoxide (DMSO) to dissolve the formazan crystals and further incubated for 10 min at 37 °C. The absorbance was determined by microplate spectrophotometer (Thermo Scientific™ Multiskan™ GO) at 570 nm.

### 2.5. Evaluation of Cell Viability via Aspartate Aminotransferase (AST) Enzyme Activity

The AST kit (Diagnosticum Zrt, Budapest, Hungary) was used to determine AST enzyme activity according to the manufacturer’s instructions. Briefly, after treatment, supernatant samples were taken from the cells. After adding the reagent to the supernatant samples, the plate was incubated for 1 min at 37 °C; then, the absorbance was repeatedly determined by microplate spectrophotometer (Thermo Scientific™ Multiskan™ GO) at 340 nm for 3 min.

### 2.6. Reverse Transcription and Real-Time PCR Analysis

Total RNA was isolated from using innuPREP RNA Mini Kit (Analytik Jena, Jena, Germany). Reverse transcription was accomplished using a RevertAid First-Strand cDNA Synthesis Kit (Thermo Scientific™) following the manufacturer’s guidelines and protocol. cDNA amplification has been done by a Real-Time PCR System (Thermo Scientific™ PikoReal™) and Sensifast™ SYBR^®^ No-ROX Kit (Bioline, London, UK).

The following primers were used:

- For CYP2E1 cDNA:
  ○ fw: 5′-AAGCAACCCGAGACACCATT-3′
  ○ rv: 5′-ACACACTCGTTTTCCTGTGG-3′;
- For RIPK1 cDNA:
  ○ fw: 5′-CGGCCTTGCCTCCTTTAAGA-3′
  ○ rv: 5′-CCGACTTCTCTGTGGGCTTT-3′;
- For RIPK3 cDNA:
  ○ fw: 5′-GCCCCAGAAGTCACTCCATC-3′
  ○ rv: 5′-AGCCCCACTTCCTATGTTGC-3′
  ○ and fw2: 5′-CATGGAGAACGGCTCCTTGT-3′
  ○ rv2: 5′-GGTTCTGGTCGTGCAGGTAA-3′.

For normalization, the simultaneous amplification of GAPDH cDNA was accomplished with the forward primer 5′-TCGGAGTCAACGGATTTGGT-3′ and reverse primer 5′-TTCCCGTTCTCAGCCTTGAC-3′ [36].

### 2.7. Measurement of Viable Cell Number Using Flow Cytometry

After treatment, the culture medium was discarded, the cells were washed twice with PBS, trypsinized, and resuspended in HBSS (Hanks’ Balanced Salt Solution, Sigma-Aldrich^®^). A suitable volume from the cell suspension supplemented with propidium iodide (PI) dye (with 10 μg/mL final concentration) was used for the determination of viable cell number using the CytoFLEX (Beckman Coulter, Brea, CA, USA) Flow Cytometer. The emission of PI was measured on the ECD channel (610/20 nm). Data were analyzed using FlowJo^®^ software.

### 2.8. Isolation and Quantitation of Protein Samples

Cells were treated as described above and were lysed in RIPA protein isolation buffer (150 mM NaCl, 1% NP-40, 50 mM Tris pH 8,0) supplemented with 1% protease inhibitor cocktail (Sigma-Aldrich ^®^), 1% phosphatase inhibitor cocktail (Sigma-Aldrich ^®^), and 1 mM PMSF. Samples were incubated on ice for 30 min and centrifuged at 14,000× *g* for 15 min at 4 °C. The supernatant was used for protein analysis and stored at −80 °C. Protein samples were quantified using the Pierce™ BCA Protein Assay Kit (Thermo Scientific™) according to the manufacturer’s guidelines.

### 2.9. Western Blot

SDS-PAGE was done by using Cleaver Scientific (Rugby, UK) omniPAGE system. Proteins were transferred onto Millipore 0.45 μm nitrocellulose membrane. Immunoblotting was performed using TBS Tween (0.1%), containing 5% non-fat dry milk for blocking membrane and 1% non-fat dry milk for antibody solutions. Loading was controlled by developing membranes for β-actin or GAPDH. The following antibodies were applied: Rabbit PolyAb Anti-PARPI (Proteintech^®^, Rosemont, IL, USA, 13371-1-AP), Rabbit PolyAb Anti-RIPK1 (Proteintech^®^, 17519-1-AP), Rabbit PolyAb Anti-RIPK3 (Proteintech^®^, 17563-1-AP), Anti-P-c-Jun (Cell Signaling, Danvers, MA, USA, 9261S), Anti-c-Jun (Cell Signaling, 9165S), and Anti-GAPDH (Santa Cruz Biotechnology, Dallas, TX, USA, 6C5). Rabbit PolyAb Anti-ACTB (Proteintech^®^, 20536-1-AP), antiHRP-conjugated secondary antibodies: HRP-Goat Anti-Rabbit IgG (Proteintech^®^, 00001-2), HRP-linked Anti-Mouse IgG (Proteintech^®^, 7076S). The bands were visualized using a chemiluminescence detection kit (Thermo Scientific™, 32,106) and VWR™ (Radnor, PA, USA) Imager Chemi Premium gel documentation system with VWR™ Image Capture Software (version: 1.6.1.0). For densitometry analysis, Western blot data were acquired using ImageJ software bundled with 64-bit Java 1.8.0_172.

### 2.10. Determination of Caspase-3/7 Activation

Cells were treated and prepared as described above. First, 3 × 10^5^ (HepG2) or 4 × 10^5^ (HepaRG) cells were centrifuged at 300 g for 5 min. Cells were resuspended in 50 μL of assay buffer (20 mM HEPES, pH 7.4, with 1% CHAPS, 5 mM DTT, and 2 mM EDTA) and stored at −80 °C for 2–3 days. After thawing, the lysates were supplemented with 17 nM Ac-DEVD-AMC (a fluorogenic substrate of caspase-3/7 proteases). The mixture was incubated at 37 °C for 1 h, and the fluorescence was determined by a fluorescent plate reader (Varioskan LUX, excitation: 380 nm, emission: 445 nm). The results were normalized to the protein content of the sample that was determined by Thermo Scientific™ Pierce™ BCA Protein Assay Kit, according to the manufacturer’s instructions.

### 2.11. GSH Measurement

For the determination of cellular GSH, monochlorbimane (mClB) derivatization followed by HPLC separation and fluorescent detection was used [37,38]. First, 10^5^ trypsinized cells in HBSS (Hanks’ Balanced Salt Solution, Sigma-Aldrich^®^) were diluted in Tris buffer (20 mM, pH 8.0) up to 100 ul, which was supplemented with 1 U/mL glutathione-S-transferase enzyme (GST) and mClB to reach 1 mM final concentration. After a 15 min incubation in the dark at RT, the derivatization was stopped with the addition of 100% trichloroacetic acid (TCA). The solution was centrifuged at 15,000× *g* for 10 min, and the supernatant was used for GSH determination. For separation, a Waters Acquity UPLC H-Class system was used, equipped with an Acquity UPLC BEH C18 2.1 × 50 mm column with an average particle diameter of 1.7 μm. Gradient elution was used as 0.25% sodium-acetate (pH 3.5) and methanol. The detector was a Waters Acquity FLR fluorescent detector with excitation and emission set to 395 and 477 nm, respectively. Quantitation was achieved by measuring GSH standards.

### 2.12. Visualization of Cell Viability, Caspase-3/7 Activity, Reduced Glutathione, and Hepatocytes (of HepaRG) by Fluorescent Microscopy

Cells were examined during and after treatments with a Nikon™ Eclipse TS2R microscope using a 4x/10x/20x phase contrast objective and a Nikon™ DS-Ri2 camera.

For visualization of cell death/viability, Hoechst 33342 (5 μg/mL) and PI dye (10 μg/mL) were added to the medium, and the cells were incubated for 30 min and for 5 min (respectively) at 37 °C. The emission of PI was examined on the TRITC channel (579–640 nm), and of Hoechst 33342 on the DAPI channel (375/28) of a Nikon™ Eclipse TS2R microscope with a Nikon™ Intensilight Epi-fluorescence Illuminator light source and a Nikon™ DS-Ri2 camera.

For visualization of caspase-3/7 activity, medium was supplemented with CellEvent™ Caspase-3/7 Green Detection Reagent (Thermo Fisher Scientific, Invitrogen™) to reach 5 μM final concentration, and the cells were incubated for at least 30 min at 37 °C. The emission of the reagent was examined on the FITC channel (516–556 nm) of a Nikon™ Eclipse TS2R microscope.

Live imaging of intracellular reduced glutathione was measured by labeling the cells with ThiolTracker™ Violet (Invitrogen™) at a final concentration of 20 μM for 30 min at 37 °C. The emission of the reagent was examined on the DAPI channel (375/28 nm) of a Nikon™ Eclipse TS2R microscope.

For the HepaRG cell line, immunofluorescent staining was used to distinguish between epithelial-like and hepatocyte populations in differentiated cells. β-catenin and E-cadherin proteins appear in the HepaRG cell line only on the surface of mature hepatocyte cells [30,35]. Cells were first washed with PBS and then fixed in −10 °C methanol for 5 min. Then, it was blocked in PBS containing 2% BSA for 30 min at RT, after which the cells were washed with PBS and labeled for 1.5 h at RT using the Anti-E-cadherin Antibody (G-10) Alexa Fluor^®^ 488: sc-8426 (Santa Cruz Biotechnology) at a concentration of 1.33 ug/1 mL PBS and Anti-β-catenin Antibody (15B8) Alexa Fluor^®^ 488 sc-53483 (Santa Cruz Biotechnology) at a concentration of 1.33 ug/1 mL PBS. After washing with PBS, the emission of conjugated antibody was examined on the FITC channel (516–556 nm) of a Nikon™ Eclipse TS2R microscope.

### 2.13. Statistical Analyses

All statistical analyses (one-way ANOVA or nonparametric Kruskal–Wallis ANOVA and Median Test) were carried out using TIBCO^®^ (Palo Alto, CA, USA) Statistica™ program (version: 13.5.0.17). *p* values were calculated with Dunnett’s test (after one-way ANOVA) or multiple comparisons (after Kruskal–Wallis test). LC_50_ values were determined using Graph Pad Prism (version: 8.0.1). Data are presented as mean ± SD from at least 3 independent experiments.

## 3. Results and Discussion

The use of experimental animals in pharmacology and toxicology is time-consuming, costly, and raises animal welfare issues; in addition, the predictive accuracy of animal in vivo testing for human adverse health effects is often questionable [39,40]. Furthermore, there is a growing need to reduce the use of experimental animals. In vitro cell-based models are often used to investigate preclinical hepatotoxicity. Due to differences in the toxicity response of different species, the use of human cell lines is advisable [41]. In in vitro models of primary human hepatocytes, immortalized human hepatic cell lines have been used, but they are limited regarding their viability, hepatic gene expression, and function [42]. Of the many options, three-dimensional (3D) models [19,20,21,22,23,24,25,26,27] and stem cell-derived models [43] have also become areas of significant interest. Developing appropriate toxicological model systems is not an easy task, but it will help the effectiveness of toxicological studies.

### 3.1. Acetaminophen Sensitivity of HepG2 and Differentiated HepaRG

HepG2 and HepaRG cell lines were used in our experiments. Both of them are of hepatic origin; however, their retention of hepatic function is markedly different. Liver-specific enzymes metabolize APAP through sulfation, glucuronidation, and to a lesser extent, hydroxylation [44]. The latter reaction is catalyzed by various isoforms of CYP450s and results in the formation of the reactive metabolite NAPQI. At high APAP doses, NAPQI depletes glutathione and forms protein adducts, resulting in the diminished activity of specific enzymes, oxidative stress, and ultimately hepatocyte death [44].

**Figure 1:**
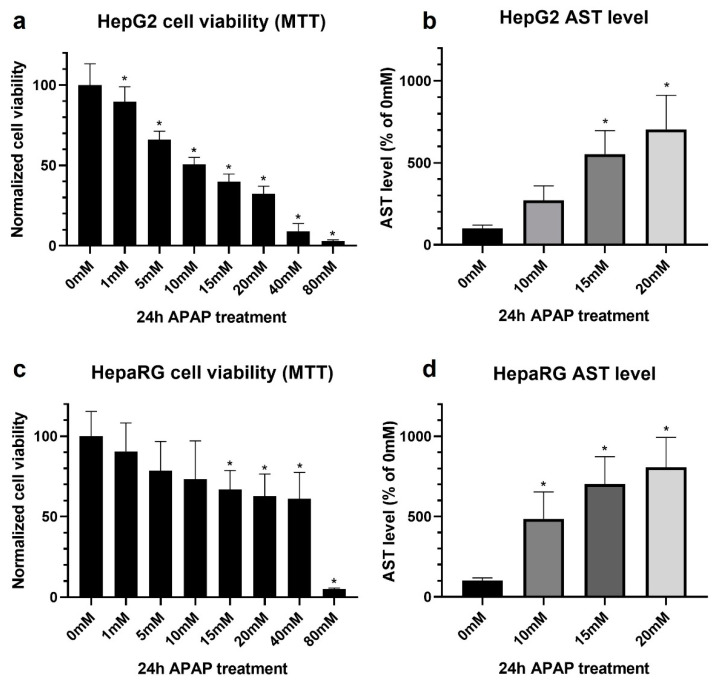
Comparison of cell viability results obtained with the MTT assay (**a**,**c**) and aspartate aminotransferase (AST) assay (**b**,**d**) using defined acetaminophen concentrations (untreated = 0 mM, 1–80 mM). Monolayer cultured HepG2 (**a**,**b**) and differentiated HepaRG (**c**,**d**) cells after 24 h of acetaminophen exposure. Data are normalized to untreated, and each data point represents the average ± SD of at least three independent experiments. * significantly different (*p* < 0.05) from untreated.

We wanted to investigate the degree of liver-specific characteristics of HepG2 and differentiated HepaRG lines via the extent of APAP-induced hepatotoxicity. Thus, both cell lines were treated with increasing concentrations of the drug; then, the cell viability was determined by MTT assay (Figure 1, left panels) and by the release of an intracellular hepatocyte-specific enzyme, aspartate aminotransferase (AST) (Figure 1, right panels). Among the liver injury markers, aminotransferases (AST, ALT) are the most commonly used in both clinical diagnosis and research involving hepatocyte damage [45].

Although the MTT assay is widely used to assess the cytotoxic potential of different compounds, our results revealed that it underperformed in the case of HepaRG cells. The MTT assay in HepG2 resulted in a toxicity profile in accordance with our expectations and previous observations [46,47]. The LC50 was found to be 10 mM (Figure 1a, Appendix B, left panel).

**Figure 2:**
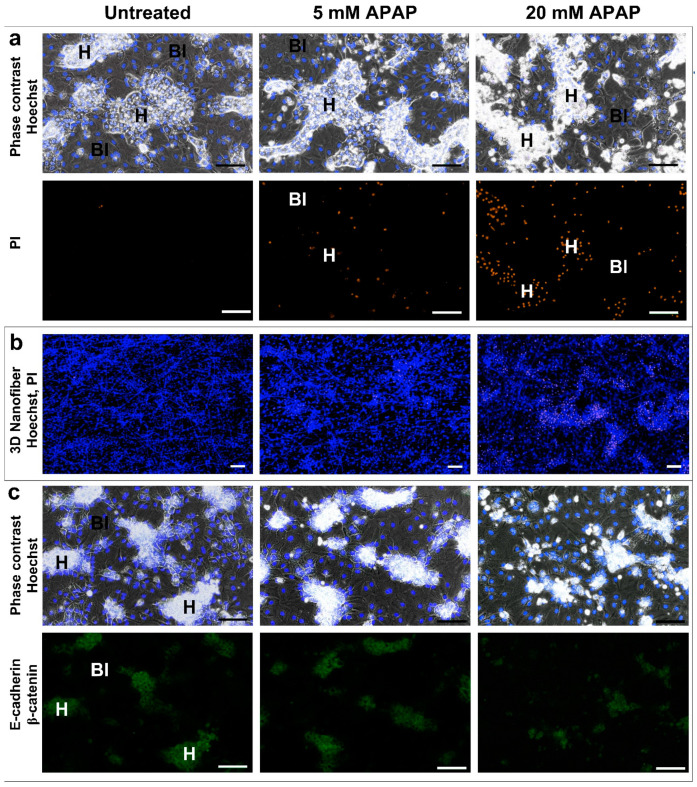
Fluorescence microscopy indicates that 24 h low-dose (5–20 mM) APAP treatment causes the selective death of hepatocytes in HepaRG cultures (H—hepatocytes, Bl—biliary-like cells). PI—positivity (orange) as a characteristic cell death nuclear staining appears exclusively in hepatocyte islands in both 2D and 3D (**a**,**b**). Immunostaining of mature hepatocytes for E-cadherin and β-catenin (green) also confirms that characteristic hepatocyte/biliary-like phenotype in control cultures is progressively lost with increasing APAP concentration (**c**). Hoechst nuclear counter stain, blue. Scale bars: 100 µm.

On the other hand, in HepaRG cultures, the toxicity could be found biphasic: a first, more sensitive phase between 1 and 20 mM and a second phase between 20 and 80 mM of APAP (Figure 1c, Appendix B, right panel). This phenomenon was also supported by fluorescence microscopy: lower APAP concentrations (first phase) resulted in marked cell death, which was limited exclusively to hepatocyte islets, whereas biliary epithelial-like cells are resistant to APAP in this concentration range (Figure 2a,b). Immunfluorescent staining was also used to distinguish between non-parenchymal biliary epithelial-like cells and hepatocytes (Figure 2c). β-catenin and E-cadherin proteins appears in the HepaRG cell line only on the surface of mature hepatocytes [30,35]. Immunostaining also supported the reduction of hepatocyte islands at 20 mM APAP (Figure 2c). Thus, the survival of non-parenchymal biliary epithelial-like cells at low APAP concentrations (up to 20 mM) masked hepatocyte-specific death assessed by MTT assay. However, the very high APAP concentration (80 mM) is toxic for the non-parenchymal biliary epithelial-like cells, too (due to nonspecific reasons such as hyperosmolarity).

By monitoring AST release (Figure 1b,d), APAP toxicity could be measured in both HepG2 and differentiated HepaRG cultures, and increased sensitivity toward hepatocyte-specific cell death was achieved in the latter case. Although both cell lines show a comparable level of AST activity, in HepaRG, the enzyme activity originates from only a fraction of the population, as hepatocytes constitute approximately 50% of the cells. Thus, the application of the determination of AST activity in non-solely hepatocyte-containing cultures as a cell death (viability) marker is suggested.

### 3.2. Investigation of Cell Death Pathways by Acetaminophen Cytotoxicity in HepG2 and Differentiated HepaRG

The nature and exact mechanism of programmed cell death pathways involved in APAP toxicity in different in vitro and in vivo models are still under investigation. Apoptosis, necroptosis, and ferroptosis are all considered to play a role either distinctly or in parallel (apoptosis and necroptosis) [8,9,48,49,50]. Furthermore, the cell death pathways involved in APAP-induced hepatocyte cell death are characteristic [8]. Through their characteristic combination, the investigated in vitro and in vivo models can be qualified.

**Figure 3:**
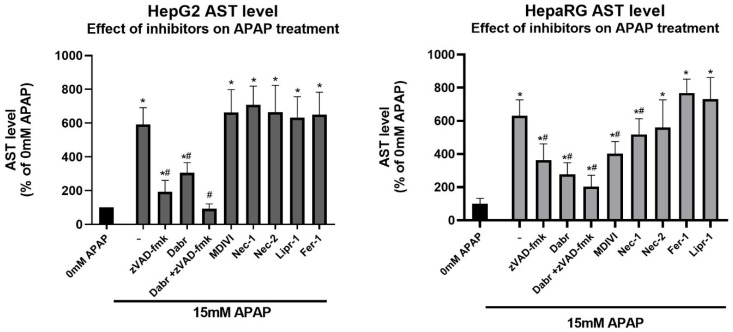
The potential effect of inhibitors in alleviating cell death induced by 15 mM acetaminophen. Monolayer cultured HepG2 (**left**) and differentiated HepaRG (**right**) cells after 24 h of acetaminophen exposure in the presence or absence of inhibitors of interest (-: vehicle (DMSO) control, Dabr: dabrafenib, Nec: necrostatin, Fer-1: ferrostatin-1, Lipr-1: liproxstatin-1). The concentrations of the inhibitors are presented in Appendix A, Table A1. Data are normalized to untreated, and each data point represents the average ± SD of at least three independent experiments. * significantly different (*p* < 0.05) from untreated (0 mM acetaminophen); # significantly different (*p* < 0.05) from group control (15 mM acetaminophen + vehicle-treated).

In order to investigate the programmed cell death initiated by APAP in our experimental setup, we assessed the potential of known specific cell death inhibitors in alleviating cytotoxicity. HepG2 and differentiated HepaRG cells cultured in a monolayer were treated with 15 mM APAP in the presence and absence of various cell death inhibitors; then, cell death was measured by the AST assay (Figure 3).

**Figure 4:**
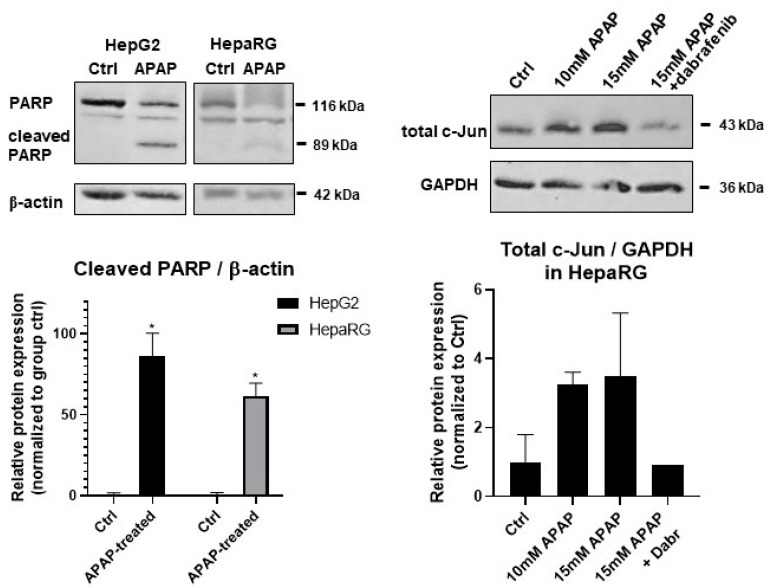
Western blot analysis of total protein samples for Poly (ADP-ribose) polymerase 1 (PARP) cleavage from monolayer cultured HepG2 and differentiated HepaRG in response to acetaminophen (APAP) treatment after 24 h (**top left** panel). Total c-Jun protein levels were determined in monolayer cultured differentiated HepaRG after 10 and 15 mM acetaminophen treatment or 15 mM acetaminophen and dabrafenib (10 μM) (**top right** panel). β-actin and GAPDH were labeled for loading control. Densitometry data represent the intensity of cleaved PARP normalized for β-actin and total c-Jun normalized to GAPDH. For each of the experiments, at least three independent measurements were carried out. * significantly different (*p* < 0.05) from untreated.

Our results show that the pan-caspase inhibitor zVAD-fmk [28] and dabrafenib [51] significantly protected both cell lines from APAP-induced cell death (Figure 3). Since distinct molecular pathways govern caspase-dependent apoptosis and necroptosis, the activation of characteristic proteins was also investigated. The caspase-mediated cleavage of the 113 kDa nuclear enzyme Poly (ADP-ribose) polymerase 1 (PARP-1) to an 89 and a 24 kDa fragment is a known hallmark of apoptosis. PARP-1 cleavage was determined in APAP-treated HepG2 and HepaRG cells (Figure 4, left panels). The 89 kDa cleaved PARP1 fragment appeared in both cell lines upon APAP treatment but more markedly in HepG2 (Figure 4, left panels).

What can be in the background of the effect of dabrafenib in the alleviation of the hepatotoxic effect of APAP?

Since RIPK3 was considered to play a key role in APAP-induced hepatotoxicity [49] and dabrafenib showed strong inhibition on RIPK3 [51], our first thought was that dabrafenib inhibited RIPK3 in our HepaRG cultures, too. However, we could not find any RIPK3 expression neither at mRNA nor at protein levels in our cultures (data not shown). Furthermore, the role of RIPK3 in APAP-induced hepatotoxicity has been the matter of intense debate [52]. Hence, the inhibitory role of dabrafenib on RIPK3 must be ruled out. At the same time, sterile-alpha motif and leucine zipper containing kinase (ZAK), a member of the MAP3K family, is known to be involved in apoptosis [53]. The overexpression of ZAK could induce apoptosis in human OS cells [53]. Furthermore, it was found that the bioflavonoid fisetin could upregulate the expression of ZAK that mediated the activation of the downstream JNK/ERK pathway, thus triggering cell apoptosis in an AP-1-dependent manner in human OS cells [53]. It was also described previously that dabrafenib inhibited ZAK kinase [54].

Thus, the c-Jun N-terminal kinase (JNK) activated total c-Jun was determined in the presence/absence of dabrafenib and APAP by Western blot (Figure 4, right panels). Indeed, an increase in total c-Jun was found by increasing APAP concentrations, which could be diminished by dabrafenib treatment to levels below those of the untreated samples. In addition, ZAK is expressed most prominently in liver, it signals to JNK through MKK4 [54], and MKK4 is the major MAP2K, which activates JNK in acute liver injury [55]. Furthermore, SIRT2-mediated deacetylation favors the phosphorylation of JNK by MKK4. Hence, it was not surprising that SIRT2-KO mice exhibited increased acetylation of JNK, which was associated with significantly reduced JNK activity in the liver. Consequently, SIRT2-KO mice showed lower cell death, minimal degenerative changes, improved liver function, and survival following APAP treatment [56]. All these observations together with our results may support the finding that dabrafenib can exert its hepatoprotective effect through the inhibition of ZAK and the following JNK pathway. Although the hallmark of RIPK-dependent necrosis, RIPK1 phosphorylation was shown by APAP treatment, and RIPK1 inhibition decreased reactive oxygen species (ROS) levels produced in APAP-injured hepatocytes in an animal model [57]; furthermore, the RIPK1 inhibitor Nec-1 also decreased the rate of hepatotoxicity in primary mice hepatocytes [9]. Both Nec-1 and Nec-2 showed no effect on our HepG2 cells and had only marginal beneficial effects in our HepaRG cultures (Figure 3). APAP treatment of both HepG2 and HepaRG resulted in PARP cleavage and the appearance of the 89 kDa fragment, further supporting the involvement of apoptosis (Figure 4, left panels).

Aside fromthe differences in hepatocyte function, the observations that (1) known specific inhibitors of necroptosis (necrostatin-1 and MDIVI) were only effective in differentiated HepaRG and (2) the degree of protection of zVAD-fmk was higher in HepG2 than in HepaRG suggest a differential execution of activated pathways. Specific inhibitors of ferroptosis (ferrostatin-1 and liproxstatin-1) were ineffective against cell viability loss in both cell lines, on the contrary of their effectiveness using in vitro and in vivo mouse models [9,10]. On the basis of all these observations, it seems HepaRG stands somewhere between HepG2 and primary hepatocytes from the hepatocyte functional point of view. However, it should be noted that the maintenance of HepaRG has some advantages over the maintenance of primary hepatocytes. A major drawback of primary hepatocytes is their limited lifespan. Isolated and in vitro cultured hepatocytes do not expand and gradually dedifferentiate, resulting in the loss of their liver-specific functions [58,59]. Hence, primary hepatocytes can only be used for a few days, making them unsuitable for long-term experiments. Primary human hepatocytes (PHHs) are considered to be the gold standard to study drug-induced liver injury, but because PHHs are usually isolated from whole livers or resected liver tissue [60], the availability of these cells is limited, and due to the interindividual variability between donors, they can differ significantly in drug response [32,61,62,63]. On the other hand, HepaRG cells are relatively immortal, and they have a stable phenotype and CYP450 expression over time. These properties of HepaRG allow us to grow identical cells in virtually unlimited amounts [32,61,64].

### 3.3. Caspase Activity and GSH Level in APAP-Treated HepG2 and Differentiated HepaRG Cells

**Figure 5:**
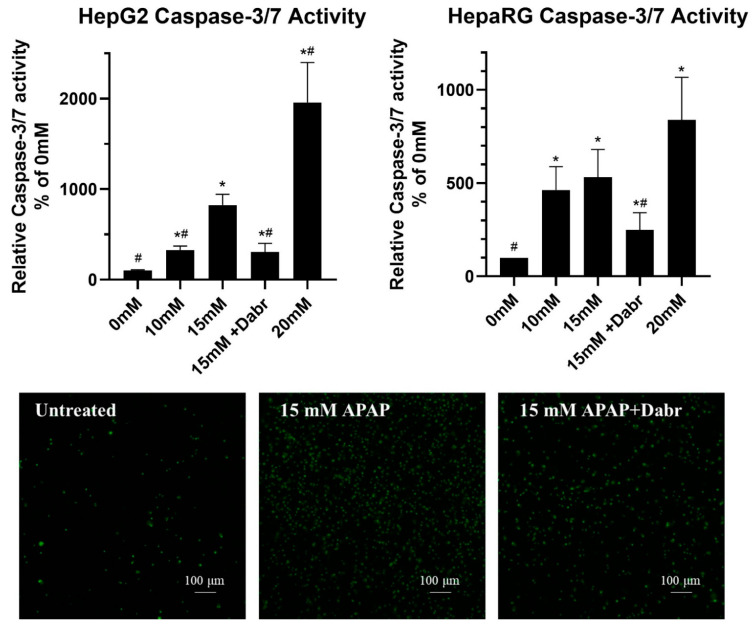
Caspase 3/7 activity induced by different concentrations of acetaminophen (0 mM—untreated, 10 mM, 15 mM and 20 mM) with or without the inhibitor dabrafenib (Dabr, 10 μM) in monolayer cultured HepG2 and differentiated HepaRG (**top** graphs). Live imaging of caspase 3/7 activity induced by 15 mM acetaminophen treatment in the presence or absence of dabrafenib (Dabr, 10 μM) after 24 h exposure, which was measured by the fluorogen substrate CellEvent in monolayer cultured differentiated HepaRG (**bottom** images). Data are normalized to untreated (0 mM), and each data point represents the average ± SD of at least three independent experiments. * significantly different (*p* < 0.05) from untreated (0 mM acetaminophen); # significantly different (*p* < 0.05) from group control (15 mM acetaminophen + vehicle-treated).

The pan-caspase inhibitor zVAD-fmk was able to protect both HepG2 and differentiated HepaRG from APAP-induced cell death (Figure 3). Thus, caspase activation was also investigated. Both cell lines were cultured in a monolayer and treated with increasing concentrations of APAP or 15 mM APAP in the presence or absence of an inhibitor. Caspase 3/7 activity was determined by fluorimetry (Figure 5, top graphs) [65]. We also investigated real-time caspase activation by a fluorescently labeled caspase substrate using fluorescence microscopy (Figure 5, bottom images).

APAP-induced caspase activation was concentration-dependent in both cell lines, further supporting the role of apoptotic mechanisms. As it could be expected, the presence of dabrafenib significantly decreased caspase activity. In parallel, an increase of the fluorogenic caspase 3/7 substrate CellEvent™ was observed in HepaRG, which could be inhibited by dabrafenib. This observation further reinforces our above detailed assumption on the possible role of dabrafenib in the inhibition of apoptosis via its inhibitory role on ZAK [54].

From the methodological perspective, it should be emphasized that to assess the degree of caspase activation in the HepaRG culture correctly, incorporating both cells and cellular fragments/debris was crucial; otherwise, cellular structures found to be positive for caspase activity could be easily lost during washing steps.

**Figure 6:**
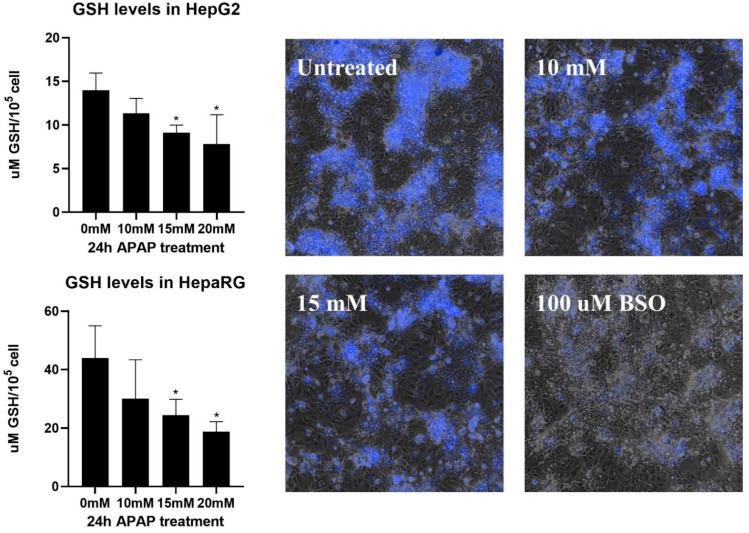
Depletion of intracellular reduced glutathione (GSH) induced by different concentrations of acetaminophen (0 mM—untreated, 10 mM, 15 mM, and 20 mM) in monolayer cultured HepG2 and differentiated HepaRG (**left** graphs). Measured glutathione concentrations were normalized to 10^5^ live cells, and each data point represents the average ± SD of at least three independent experiments. * significantly different (*p* < 0.05) from untreated (0 mM acetaminophen). Live imaging of intracellular reduced glutathione levels after acetaminophen treatment (0 mM—untreated, 10 mM, and 15 mM) after 24 h exposure, measured by the fluorescent dye ThiolTracker™ Violet in monolayer cultured differentiated HepaRG (**right** images).

Conjugation with glutathione is an important moment of hepatic APAP metabolism [44]. At lower doses, APAP biotransformation proceeds without physiological disturbance; however, higher doses cause glutathione depletion, which leads to oxidative stress and oxidative damage, initiating signaling pathways that can drive the cell to programmed cell death [44]. Consequently, the level of reduced cellular glutathione is a suitable marker for monitoring APAP metabolism in hepatocytes. Therefore, the reduced form of cellular glutathione was determined in monolayer cultured HepG2 and differentiated HepaRG (Figure 6).

Glutathione decreased in both cell lines, with a more pronounced decrease seen in HepaRG since 15 mM APAP halved the cellular reduced glutathione pool. This observation highlights again that HepaRG has kept its hepatic function to a greater extent than HepG2, and it is more suitable for toxicological studies. It is also important to emphasize that normalization of the measured glutathione by cell count or protein concentration can bias the results toward surviving biliary epithelial-like cells. In order to visualize the differential depletion of glutathione among the cell types present in differentiated HepaRG culture, we labeled APAP-treated cells with a thiol-tracking probe (Figure 6, right images).

Live cell fluorescent imaging revealed intensive labeling of hepatocyte islets in untreated cells (Figure 6, right images), which consistently with the hepatic phenotype contain the highest concentration of cellular glutathione among mammalian cells [66,67]. Glutathione within hepatocyte islets showed a proportional decrease with increasing APAP concentrations and approached that achieved by buthionine sulfoximine (BSO) depletion. These observations further confirm the hepatocyte-mediated metabolism of APAP and the accompanying reduction of cellular glutathione.

### 3.4. The Effect of 3D Culture Techniques (Spheroid and Nanofiber) on Acetaminophen Cytotoxicity in HepG2 and Differentiated HepaRG Cells

The efficient metabolism of APAP corresponds to the level of phase I enzymes in hepatocytes. Most frequently, the dominating role in the conversion of APAP to the highly reactive metabolite NAPQI is ascribed to the isoform CYP2E1 [28,68]. HepG2 and differentiated HepaRG are known to possess a different degree of hepatic functions; this difference also expands to the level of enzymes involved in the process of drug metabolism [69]. Developments in cell culture techniques aim at narrowing the gap between in vitro and in vivo models. Regarding hepatic in vitro models, 3D culture methods are extensively used to increase hepatic function [19,20,21,22,23,24,25,26,27].

**Figure 7:**
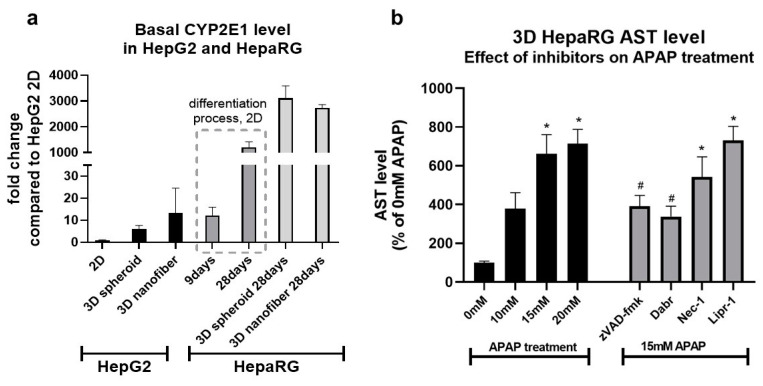
The effect of 3D culture techniques (spheroid and nanofiber) on CYP2E1 mRNA expression and APAP-induced cell death in HepG2 and differentiated HepaRG (**a**). CYP2E1 mRNA was determined by real-time RT-PCR from both cell lines cultured either in a 2D monolayer or 3D (spheroid or nanofiber). Each CYP2E1 expression is normalized to the expression of the 2D cultured HepG2 line. In the case of HepaRG, CYP2E1 was also monitored throughout the differentiation process (9 and 28 days). Cell death induced by different concentrations of acetaminophen (APAP, 0 mM—untreated, 10 mM, 15 mM, and 20 mM) or 15 mM acetaminophen and inhibitors (zVAD-fmk 40 μM, dabrafenib (Dabr) 10 μM, necrostatin-1 (Nec-1) 50 μM, or liproxstatin-1 (Lipr-1) 1 μM) was measured by the AST assay (**b**). Data are normalized to untreated (0 mM), and each data point represents the average ± SD of at least three independent experiments. * significantly different (*p* < 0.05) from untreated (0 mM APAP); # significantly different (*p* < 0.05) from 15 mM APAP.

We aimed at the investigation of the effect of two commonly used 3D culture methods—spheroid and nanofiber culture—on the hepatic function and APAP sensitivity of HepG2 and HepaRG. Cells were cultured in 3D by two methods as shown, and CYP2E1 mRNA was quantified; in the case of HepaRG, the level of CYP2E1 was also measured throughout the differentiation process (Figure 7, upper panels).

Regarding 2D culture, undifferentiated HepaRG expressed 10 times more CYP2E1 mRNA than HepG2, and it increased further by 100-fold (Figure 7a) at the end of the differentiation.

By applying 3D culture methods, CYP2E1 mRNA levels could also be elevated. In HepG2, nanofiber culture resulted in the most robust increase, while in differentiated HepaRG, both 3D methods induced CYP2E1 expression to a similar degree (Figure 7a). Despite the effectiveness achieved by the nanofiber method in HepG2, the resulting mRNA levels were closest to those measured in undifferentiated HepaRG (9 days, Figure 7a). The elevated level of CYP2E1 mRNA in 3D cultured HepG2 could also be the result of prolonged cultivation, as HepG2 grown for 21 days in 2D monolayer showed a similar increase in hepatic function [70].

Based on the effectiveness in the induction of CYP2E1 by 3D culture—observed most prominently in HepaRG—we were interested in whether it also increases APAP sensitivity. Thus, HepaRG was differentiated on nanofiber plates and treated with different concentrations of APAP. To investigate for a deviation in the cytotoxic profile, cells were also incubated in the presence or absence of inhibitors of interest (Figure 7b).

APAP increased AST release in a dose-dependent manner; however, the extent was not significantly different compared to 2D cultured differentiated HepaRG. The applied inhibitors were also protective to a similar degree, supposing no difference in sensitivity nor the cytotoxic profile despite increased levels of CYP2E1.

Summarily, the hepatic functions of HepaRG stand closer to those of primary hepatocytes, but the 3D cultivation, especially in APAP toxicity studies, is not necessarily worth the more complicated and expensive maintenance.

## 4. Conclusions

The investigation of drug-induced hepatotoxicity and the prediction of clinical drug-induced liver damage requires appropriate in vivo and in vitro toxicological model systems. In our study, an attempt was made to compare different three-dimensional and stem cell-derived models to find the most appropriate one.

The degree of liver-specific characteristics of HepG2 and differentiated HepaRG lines via the extent of APAP-induced hepatotoxicity was followed.

The MTT assay in HepG2 resulted in a toxicity profile in accordance with our expectations and previous observations. However, our results revealed that it underperformed with HepaRG. In the case of the HepaRG line, the survival of non-parenchymal biliary epithelial-like cells at low APAP concentrations masked hepatocyte-specific death assessed by MTT assay. Thus, the application of the determination of AST activity in non-solely hepatocyte containing cultures as a cell death (viability) marker is suggested.

Since the pathways involved in APAP-induced hepatocyte cell death are characteristic, the investigated in vitro and in vivo models can be qualified by them. Thus, the effect of known specific cell death inhibitors and the activation of characteristic proteins was investigated. The pan-caspase inhibitor zVAD-fmk and dabrafenib significantly protected both cell lines from APAP-induced cell death. The differences in hepatocyte function and the observations that (1) known specific inhibitors of necroptosis (necrostatin-1 and MDIVI) were only effective in differentiated HepaRG and (2) the degree of protection of zVAD-fmk was higher in HepG2 than in HepaRG suggest a differential execution of activated pathways. On the basis of all these observations, it seems HepaRG stands somewhere between HepG2 and primary hepatocytes from the hepatocyte functional point of view. However, it should be noted that the maintenance of HepaRG has more advantages over the maintenance of primary hepatocytes such as relative immortality, stable phenotype, and CYP450 expression. These properties of HepaRG allow us to grow identical cells in virtually unlimited amounts.

By applying 3D culture methods, CYP2E1 mRNA levels could also be elevated. In HepG2, nanofiber culture resulted in the most robust increase, while in differentiated HepaRG, both 3D methods induced CYP2E1 expression to a similar degree. Albeit APAP treatment increased the AST release in a dose-dependent manner of 3D cultured HepaRG cells, it was not significantly different compared to 2D cultured differentiated HepaRG.

Summarily, the hepatic functions of HepaRG stand closer to those of primary hepatocytes, but the 3D cultivation, especially in APAP toxicity studies, is not necessarily worth the more complicated and expensive maintenance.

## Figures and Tables

**Figure A1:**
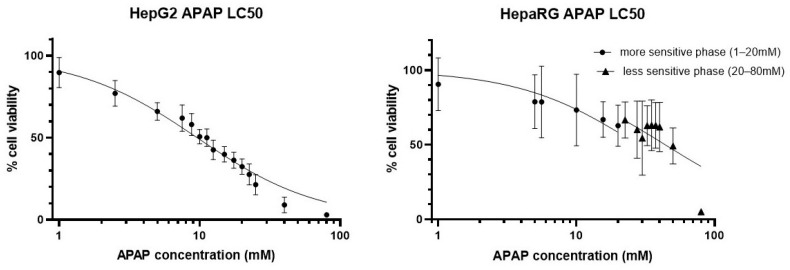
Cell viability of monolayer cultured HepG2 (**left**) and differentiated HepaRG (**right**) cells after 24 h of acetaminophen exposure measured by the MTT-assay. Data points are normalized to untreated, and each data point represents the average ± SD of at least three independent experiments. Logistic curves were fitted using Graph Pad Prism 8 software.

## Data Availability

The data presented in this study are available on request from the corresponding author. The data are not publicly available due to the Material Transfer Agreement of the University of Technology and Economics and Inserm Transfert SA on the HepaRG cell line.

## Appendix A

**Table A1:** Inhibitory compounds used for inhibitor profile studies.

Inhibitory Compound	Abbreviation	Mechanism of Inhibition	Final Concentration in HepG2 cells	Final Concentration in HepaRG Cells
zVAD-fmk (solved in DMSO, Selleckchem)	Z-V-f	inhibition of caspases, apoptosis	50 μM	40 μM
Dabrafenib-mesylate (solved in DMSO, MCE^®^)	Dabr	inhibition of B-RafV600E, RIPK3, ZAK kinase	10 μM	10 μM
Necrostatin-1 (solved in DMSO, Santa Cruz Biotechnology)	Nec-1	inhibition of RIPK1, necroptosis	20 μM	50 μM
Necrostatin-2 (solved in DMSO, MCE^®^)	Nec-2	inhibition of necroptosis	25 μM	50 μM
MDIVI-1 (solved in DMSO MCE^®^)	MDIVI	inhibition of mitochondrial division, necroptosis	25 μM	50 μM
Liproxstatin-1 (solved in DMSO, Sigma^®^)	Lip-1	inhibition of lipid peroxidation, ferroptosis	1 μM	1 μM
Ferrostatin-1 (solved in DMSO, Selleckchem)	Fer-1	inhibition of lipid peroxidation, ferroptosis	1 μM	10 μM

## References

[1] Weaver R.J., Blomme E.A., Chadwick A.E., Copple I.M., Gerets H.H.J., Goldring C.E., Guillouzo A., Hewitt P.G., Ingelman-Sundberg M., Jensen K.G. (2020). Managing the challenge of drug-induced liver injury: A roadmap for the development and deployment of preclinical predictive models. Nat. Rev. Drug Discov..

[2] Maes M., Vinken M., Jaeschke H. (2016). Experimental models of hepatotoxicity related to acute liver failure. Toxicol. Appl. Pharmacol..

[3] Ramachandran A., Jaeschke H. (2017). Acetaminophen toxicity: Novel insights into mechanisms and future perspectives. Gene Expr..

[4] Fontana R.J. (2008). Acute liver failure including acetaminophen overdose. Med. Clin. N. Am..

[5] Dahlin D.C., Miwa G.T., Lu A.Y., Nelson S.D. (1984). N-Acetyl-p-Benzoquinone imine: A cytochrome P-450-mediated oxidation product of acetaminophen. Proc. Natl. Acad. Sci. USA.

[6] Mitchell J.R., Jollow D.J., Potter W.Z., Gillette J.R., Brodie B.B. (1973). Acetaminophen induced hepatic necrosis. IV. Protective role of glutathione. J. Pharmacol. Exp. Ther..

[7] Rosen G.M., Rauckman E.J., Ellington S.P., Dahlin D.C., Christie J.L., Nelson S.D. (1984). Reduction and Glutathione Conjugation Reactions of N-Acetyl-p-Benzoquinone Imine and Two Dimethylated Analogues. Mol. Pharmacol..

[8] Jaeschke H., Ramachandran A., Chao X., Ding W.X. (2019). Emerging and Established Modes of Cell Death during Acetaminophen-Induced Liver Injury. Arch. Toxicol..

[9] Lőrincz T., Jemnitz K., Kardon T., Mandl J., Szarka A. (2015). Ferroptosis is involved in acetaminophen induced cell death. Pathol. Oncol. Res..

[10] Yamada N., Karasawa T., Kimura H., Watanabe S., Komada T., Kamata R., Sampilvanjil A., Ito J., Nakagawa K., Kuwata H. (2020). Ferroptosis driven by radical oxidation of N-6 polyunsaturated fatty acids mediates acetaminophen-induced acute liver failure. Cell Death Dis..

[11] Yamada N., Karasawa T., Takahashi M. (2020). Role of Ferroptosis in Acetaminophen-Induced Hepatotoxicity. Arch. Toxicol..

[12] Hamid Boulares A., Zoltoski A.J., Stoica B.A., Cuvillier O., Smulson M.E. (2002). Acetaminophen Induces a Caspase-Dependent and Bcl-XL Sensitive Apoptosis in Human Hepatoma Cells and Lymphocytes. Pharmacol. Toxicol..

[13] Kass G.E.N., Macanas-Pirard P., Lee P.C., Hinton R.H. (2003). The role of apoptosis in acetaminophen-induced injury. Annals of the New York Academy of Sciences.

[14] Manov I., Hirsh M., Iancu T.C. (2004). N-Acetylcysteine Does Not Protect HepG2 Cells against Acetaminophen-Induced Apoptosis. Basic Clin. Pharmacol. Toxicol..

[15] Chao X., Wang H., Jaeschke H., Ding W.X. (2018). Role and Mechanisms of Autophagy in Acetaminophen-Induced Liver Injury. Liver Int..

[16] Imaeda A.B., Watanabe A., Sohail M.A., Mahmood S., Mohamadnejad M., Sutterwala F.S., Flavell R.A., Mehal W.Z. (2009). Acetaminophen-Induced Hepatotoxicity in Mice Is Dependent on Tlr9 and the Nalp3 Inflammasome. J. Clin. Investig..

[17] Williams C.D., Farhood A., Jaeschke H. (2010). Role of Caspase-1 and Interleukin-1β in Acetaminophen-Induced Hepatic Inflammation and Liver Injury. Toxicol. Appl. Pharmacol..

[18] Xie Y., McGill M.R., Dorko K., Kumer S.C., Schmitt T.M., Forster J., Jaeschke H. (2014). Mechanisms of Acetaminophen-Induced Cell Death in Primary Human Hepatocytes. Toxicol. Appl. Pharmacol..

[19] Aritomi K., Ishitsuka Y., Tomishima Y., Shimizu D., Abe N., Shuto T., Irikura M., Kai H., Irie T. (2014). Evaluation of Three-Dimensional Cultured HepG2 Cells in a Nano Culture Plate System: An in Vitro Human Model of Acetaminophen Hepatotoxicity. J. Pharmacol. Sci..

[20] Ramaiahgari S.C., den Braver M.W., Herpers B., Terpstra V., Commandeur J.N.M., van de Water B., Price L.S. (2014). A 3D in vitro model of differentiated HepG2 cell spheroids with improved liver-like properties for repeated dose high-throughput toxicity studies. Arch. Toxicol..

[21] Ellero A.A., van den Bout I., Vlok M., Cromarty A.D., Hurrell T. (2021). Continual proteomic divergence of HepG2 cells as a consequence of long-term spheroid culture. Sci. Rep..

[22] Mandon M., Huet S., Dubreil E., Fessard V., Le Hégarat L. (2019). Three-Dimensional HepaRG Spheroids as a Liver Model to Study Human Genotoxicity in Vitro with the Single Cell Gel Electrophoresis Assay. Sci. Rep..

[23] Higuchi Y., Kawai K., Kanaki T., Yamazaki H., Chesné C., Guguen-Guillouzo C., Suemizu H. (2016). Functional Polymer-Dependent 3D Culture Accelerates the Differentiation of HepaRG Cells into Mature Hepatocytes. Hepatol. Res..

[24] Gunness P., Mueller D., Shevchenko V., Heinzle E., Ingelman-Sundberg M., Noor F. (2013). 3D Organotypic Cultures of Human HepaRG Cells: A Tool for In Vitro Toxicity Studies. Toxicol. Sci..

[25] Berger B., Donzelli M., Maseneni S., Boess F., Roth A., Krähenbühl S., Haschke M. (2016). Comparison Of Liver Cell Models Using The Basel Phenotyping Cocktail. Front. Pharmacol..

[26] Oda S., Uchida Y., Aleo M.D., Koza-Taylor P.H., Matsui Y., Hizue M., Marroquin L.D., Whritenour J., Uchida E., Yokoi T. (2021). An in Vitro Coculture System of Human Peripheral Blood Mononuclear Cells with Hepatocellular Carcinoma-Derived Cells for Predicting Drug-Induced Liver Injury. Arch. Toxicol..

[27] Chen S., Wu Q., Li X., Li D., Mei N., Ning B., Puig M., Ren Z., Tolleson W.H., Guo L. (2021). Characterization of Cytochrome P450s (CYP)-Overexpressing HepG2 Cells for Assessing Drug and Chemical-Induced Liver Toxicity. J. Environ. Sci. Health Part C Toxicol. Carcinog..

[28] Bai J., Cederbaum A.I. (2004). Adenovirus Mediated Overexpression of CYP2E1 Increases Sensitivity of HepG2 Cells to Acetaminophen Induced Cytotoxicity. Mol. Cell. Biochem..

[29] Gripon P., Rumin S., Urban S., Le Seyec J., Glaise D., Cannie I., Guyomard C., Lucas J., Trepo C., Guguen-Guillouzo C. (2002). Infection of a Human Hepatoma Cell Line by Hepatitis B Virus. Proc. Natl. Acad. Sci. USA.

[30] Cerec V., Glaise D., Garnier D., Morosan S., Turlin B., Drenou B., Gripon P., Kremsdorf D., Guguen-Guillouzo C., Corlu A. (2007). Transdifferentiation of Hepatocyte-like Cells from the Human Hepatoma HepaRG Cell Line through Bipotent Progenitor. Hepatology.

[31] Parent R., Marion M.-J., Furio L., Trépo C., Petit M.-A. (2004). Origin and Characterization of a Human Bipotent Liver Progenitor Cell Line. Gastroenterology.

[32] McGill M.R., Yan H.-M.M., Ramachandran A., Murray G.J., Rollins D.E., Jaeschke H. (2011). HepaRG Cells: A Human Model to Study Mechanisms of Acetaminophen Hepatotoxicity. Hepatology.

[33] Anthérieu S., Chesné C., Li R., Guguen-Guillouzo C., Guillouzo A. (2012). Optimization of the HepaRG Cell Model for Drug Metabolism and Toxicity Studies. Toxicol. In Vitro.

[34] Wu Y., Geng X., Wang J., Miao Y., Lu Y., Li B. (2016). The HepaRG Cell Line, a Superior in Vitro Model to L-02, HepG2 and HiHeps Cell Lines for Assessing Drug-Induced Liver Injury. Cell Biol. Toxicol..

[35] Tascher G., Burban A., Camus S., Plumel M., Chanon S., Le Guevel R., Shevchenko V., van Dorsselaer A., Lefai E., Guguen-Guillouzo C. (2019). In-Depth Proteome Analysis Highlights HepaRG Cells as a Versatile Cell System Surrogate for Primary Human Hepatocytes. Cells.

[36] Fox B.C., Devonshire A.S., Schutte M.E., Foy C.A., Minguez J., Przyborski S., Maltman D., Bokhari M., Marshall D. (2010). Validation of Reference Gene Stability for APAP Hepatotoxicity Studies in Different in Vitro Systems and Identification of Novel Potential Toxicity Biomarkers. Toxicol. In Vitro.

[37] Lőrincz T., Szarka A. (2017). The Determination of Hepatic Glutathione at Tissue and Subcellular Level. J. Pharmacol. Toxicol. Methods.

[38] Hajdinák P., Czobor Á., Lőrincz T., Szarka A. (2019). The Problem of Glutathione Determination: A Comparative Study on the Measurement of Glutathione from Plant Cells. Period. Polytech. Chem. Eng..

[39] May J.E., Xu J., Morse H.R., Avent N.D., Donaldson C. (2009). Toxicity Testing: The Search for an in Vitro Alternative to Animal Testing. Br. J. Biomed. Sci..

[40] Han W., Wu Q., Zhang X., Duan Z. (2019). Innovation for Hepatotoxicity in Vitro Research Models: A Review. J. Appl. Toxicol..

[41] Hawksworth G. (1994). Advantages and Disadvantages of Using Human Cells for Pharmacological and Toxicological Studies. Hum. Exp. Toxicol..

[42] Zeilinger K., Freyer N., Damm G., Seehofer D., Knöspel F. (2016). Cell Sources for in Vitro Human Liver Cell Culture Models. Exp. Biol. Med..

[43] Jin M., Yi X., Liao W., Chen Q., Yang W., Li Y., Li S., Gao Y., Peng Q., Zhou S. (2021). Advancements in Stem Cell-Derived Hepatocyte-like Cell Models for Hepatotoxicity Testing. Stem Cell Res. Ther..

[44] Mazaleuskaya L.L., Sangkuhl K., Thorn C.F., FitzGerald G.A., Altman R.B., Klein T.E. (2015). PharmGKB Summary: Pathways of Acetaminophen Metabolism at the Therapeutic versus Toxic Doses. Pharmacogenet. Genomics.

[45] McGill M.R. (2016). The Past and Present of Serum Aminotransferases and the Future of Liver Injury Biomarkers. EXCLI J..

[46] Jang M., Neuzil P., Volk T., Manz A., Kleber A. (2015). On-Chip Three-Dimensional Cell Culture in Phaseguides Improves Hepatocyte Functions in Vitro. Biomicrofluidics.

[47] González L.T., Minsky N.W., Espinosa L.E.M., Aranda R.S., Meseguer J.P., Pérez P.C. (2017). In Vitro Assessment of Hepatoprotective Agents against Damage Induced by Acetaminophen and CCl4. BMC Complement. Altern. Med..

[48] Zhang Y.F., He W., Zhang C., Liu X.J., Lu Y., Wang H., Zhang Z.H., Chen X., Xu D.X. (2014). Role of Receptor Interacting Protein (RIP)1 on Apoptosis-Inducing Factor-Mediated Necroptosis during Acetaminophen-Evoked Acute Liver Failure in Mice. Toxicol. Lett..

[49] Ramachandran A., McGill M.R., Xie Y., Ni H.-M., Ding W.-X., Jaeschke H. (2013). Receptor Interacting Protein Kinase 3 Is a Critical Early Mediator of Acetaminophen-Induced Hepatocyte Necrosis in Mice. Hepatology.

[50] Sharma M., Gadang V., Jaeschke A. (2012). Critical Role for Mixed-Lineage Kinase 3 in Acetaminophen-Induced Hepatotoxicity. Mol. Pharmacol..

[51] Li J.X., Feng J.M., Wang Y., Li X.H., Chen X.X., Su Y., Shen Y.Y., Chen Y., Xiong B., Yang C.H. (2014). The B-RafV600E Inhibitor Dabrafenib Selectively Inhibits RIP3 and Alleviates Acetaminophen-Induced Liver Injury. Cell Death Dis..

[52] Dara L., Johnson H., Suda J., Win S., Gaarde W., Han D., Kaplowitz N. (2015). Receptor Interacting Protein Kinase 1 Mediates Murine Acetaminophen Toxicity Independent of the Necrosome and Not through Necroptosis. Hepatology.

[53] Fu C.-Y., Chen M.-C., Tseng Y.-S., Chen M.-C., Zhou Z., Yang J.-J., Lin Y.-M., Viswanadha V.P., Wang G., Huang C.-Y. (2019). Fisetin Activates Hippo Pathway and JNK/ERK/AP-1 Signaling to Inhibit Proliferation and Induce Apoptosis of Human Osteosarcoma Cells via ZAK Overexpression. Environ. Toxicol..

[54] Vin H., Ojeda S.S., Ching G., Leung M.L., Chitsazzadeh V., Dwyer D.W., Adelmann C.H., Restrepo M., Richards K.N., Stewart L.R. (2013). BRAF Inhibitors Suppress Apoptosis through Off-Target Inhibition of JNK Signaling. eLife.

[55] Zhang J., Min R.W.M., Le K., Zhou S., Aghajan M., Than T.A., Win S., Kaplowitz N. (2017). The Role of MAP2 Kinases and P38 Kinase in Acute Murine Liver Injury Models. Cell Death Dis..

[56] Sarikhani M., Mishra S., Arumugam P., Chaithanya D., Donald K. (2018). SIRT2 Regulates Oxidative Stress-Induced Cell Death through Deacetylation of c-Jun NH 2 -Terminal Kinase. Cell Death Differ..

[57] Takemoto K., Hatano E., Iwaisako K., Takeiri M., Noma N., Ohmae S., Toriguchi K., Tanabe K., Tanaka H., Seo S. (2014). Necrostatin-1 Protects against Reactive Oxygen Species (ROS)-Induced Hepatotoxicity in Acetaminophen-Induced Acute Liver Failure. FEBS Open Bio.

[58] Hewitt N.J., Lechón M.J.G., Houston J.B., Hallifax D., Brown H.S., Maurel P., Kenna J.G., Gustavsson L., Lohmann C., Skonberg C. (2007). Primary Hepatocytes: Current Understanding of the Regulation of Metabolic Enzymes and Transporter Proteins, and Pharmaceutical Practice for the Use of Hepatocytes in Metabolism, Enzyme Induction, Transporter, Clearance, and Hepatotoxicity Studies. Drug Metab. Rev..

[59] Rowe C., Gerrard D.T., Jenkins R., Berry A., Durkin K., Sundstrom L., Goldring C.E., Park B.K., Kitteringham N.R., Hanley K.P. (2013). Proteome-Wide Analyses of Human Hepatocytes during Differentiation and Dedifferentiation. Hepatology.

[60] Kleine M., Riemer M., Krech T., DeTemple D., Jäger M.D., Lehner F., Manns M.P., Klempnauer J., Borlak J., Bektas H. (2014). Explanted Diseased Livers—A Possible Source of Metabolic Competent Primary Human Hepatocytes. PLoS ONE.

[61] Kiamehr M., Heiskanen L., Laufer T., Düsterloh A., Kahraman M., Käkelä R., Laaksonen R., Aalto-Setälä K. (2019). Dedifferentiation of Primary Hepatocytes Is Accompanied with Reorganization of Lipid Metabolism Indicated by Altered Molecular Lipid and MiRNA Profiles. Int. J. Mol. Sci..

[62] Kim Y., Kang K., Jeong J., Paik S.S., Kim J.S., Park S.A., Kim W.D., Park J., Choi D. (2017). Three-Dimensional (3D) Printing of Mouse Primary Hepatocytes to Generate 3D Hepatic Structure. Ann. Surg. Treat. Res..

[63] Nawaz A., Razpotnik A., Rouimi P., de Sousa G., Cravedi J.P., Rahmani R. (2014). Cellular Impact of Combinations of Endosulfan, Atrazine, and Chlorpyrifos on Human Primary Hepatocytes and HepaRG Cells after Short and Chronic Exposures. Cell Biol. Toxicol..

[64] Turpeinen M., Tolonen A., Chesne C., Guillouzo A., Uusitalo J., Pelkonen O. (2009). Functional Expression, Inhibition and Induction of CYP Enzymes in HepaRG Cells. Toxicol. In Vitro.

[65] Hajdinák P., Czobor Á., Szarka A. (2019). The Potential Role of Acrolein in Plant Ferroptosis-like Cell Death. PLoS ONE.

[66] Lu S.C. (2013). Glutathione synthesis. Biochim. Biophys. Acta.

[67] Summer K.H., Wiebel F.J. (1981). Glutathione and Glutathione S-Transferase Activities of Mammalian Cells in Culture. Toxicol. Lett..

[68] Manyike P.T., Kharasch E.D., Kalhorn T.F., Slattery J.T. (2000). Contribution of CYP2E1 and CYP3A to Acetaminophen Reactive Metabolite Formation. Clin. Pharmacol. Ther..

[69] Hart S.N., Li Y., Nakamoto K., Subileau E., Steen D., Zhong X. (2010). A Comparison of Whole Genome Gene Expression Profiles of HepaRG Cells and HepG2 Cells to Primary Human Hepatocytes and Human Liver Tissues. Drug Metab. Dispos..

[70] Luckert C., Schulz C., Lehmann N., Thomas M., Hofmann U., Hammad S., Hengstler J.G., Braeuning A., Lampen A., Hessel S. (2017). Comparative Analysis of 3D Culture Methods on Human HepG2 Cells. Arch. Toxicol..

